# Incidence and characteristics of adverse drug reactions in a cohort of patients treated with PD-1/PD-L1 inhibitors in real-world practice

**DOI:** 10.3389/fmed.2022.891179

**Published:** 2022-08-22

**Authors:** Mònica Sabaté Gallego, Eulàlia Pérez Esquirol, Núria Garcia Doladé, Xavier Vidal Guitart, Maria-Josep Carreras Soler, Anna Farriols Danés, Enriqueta Felip, Irene Braña, Joan Carles Galceran, Rafael Morales Barrera, Eva Muñoz-Couselo, Antònia Agustí Escasany

**Affiliations:** ^1^Clinical Pharmacology Service, Vall d'Hebron Barcelona Hospital Campus, Barcelona, Spain; ^2^Department of Pharmacology, Therapeutics and Toxicology, Universitat Autònoma de Barcelona, Bellaterra, Barcelona, Spain; ^3^Catalan Institute of Pharmacology Foundation, Vall d'Hebron Barcelona Hospital Campus, Barcelona, Spain; ^4^Pharmacy Service, Vall d'Hebron Barcelona Hospital Campus, Barcelona, Spain; ^5^Clinical Oncology Service, Vall d'Hebron Barcelona Hospital Campus, Barcelona, Spain; ^6^Vall d'Hebron Institute of Oncology, Vall d'Hebron Barcelona Hospital Campus, Barcelona, Spain

**Keywords:** immunotherapy, adverse reaction, immune-related adverse reaction, pharmacovigilance, real-world practice

## Abstract

**Background:**

Data related to adverse drug reactions (ADRs), specifically immune-related adverse events (irAEs), in long-term treatment with immunotherapy in real-world practice is scarce, as is general information regarding the management of ADRs.

**Objectives:**

To characterize and describe the incidence of ADRs in patients who began immunotherapy treatment in clinical practice.

**Methods:**

In a prospective observational study cancer patients ≥18 years of age who were treated with a monotherapy regime of PD-1/PD-L1 inhibitors were evaluated. The study period was from November 2017 to June 2019 and patients were followed up until June 2021. Patients were contacted monthly by telephone and their electronic health records were reviewed. Each ADR was graded according to the Common Terminology Criteria for Adverse Events (CTCAE 5.0).

**Results:**

Out of 99 patients, 86 met the inclusion criteria. Most were male (67.4%), with a median age of 66 (interquartile range, IQR: 59–76). The most frequent cancer was non-small cellular lung cancer (46 cases, 53.5%), followed by melanoma (22, 25.6%). A total of 74 patients (86%) were treated with anti-PD-1 drugs and 12 (14%) were treated with anti-PD-L1 drugs. The median treatment durations were 4.9 (IQR: 1.9–17.0) and 5.9 months (IQR: 1.2–12.3), respectively. Sixty-three patients (73%) developed from a total of 156 (44% of the total number of ADR) irADRs, wherein the most frequent were skin disorders (50 cases, 32%, incidence = 30.5 irADRs/100 patients per year [p-y]), gastrointestinal disorders (29, 19%, 17.7 irADRs/100 p-y), musculoskeletal disorders (17, 11%, 10.4 irADRs/100 p-y), and endocrine disorders (14, 9%, 8.6 irADRs/100 p-y). A total of 22 irADRs (14%) had a latency period of ≥12 months. Twelve irADRs (7.7%) were categorized as grade 3–4, and while 2 (1.3%) were categorized as grade 5 (death). Sixty-one irADRs (39.1%) in 36 patients required pharmacological treatment and 47 irADRs (30.1%) in 22 patients required treatment with corticosteriods.

**Conclusion:**

The majority of patients treated with anti-PD1/PDL1-based immunotherapy experienced adverse reactions. Although most of these reactions were mild, 11.5% were categorized as grade 3 or above. A high percentage of the reactions were immune-related and occurred throughout the treatment, thereby indicating that early identification and close monitoring is essential.

## Introduction

Since their initial approval by the European Medicines Agency (EMA), the use of immunotherapy drugs in different cancer indications has increased gradually. As such, there is a requirement to detect the occurrence of adverse drug reactions (ADRs), and in particular, immune-related adverse events (irAEs), when these treatments are used over a prolonged period of time in real-world clinical practice.

Immune checkpoint inhibitors (CPIs), such as the cytotoxic T lymphocyte associated antigen-4 (CTLA-4) antibody ipilimumab and the programmed cell death (PD-1)/programmed cell death ligand-1 (PD-L1) antibodies nivolumab, pembrolizumab, and atezolizumab, were the first drugs approved for use in immunotherapy to treat cancer (1–3). More specifically, in May 2017, nivolumab and pembrolizumab were approved for some indications, such as advanced melanoma, non-small cell lung cancer, Hodgkin's lymphoma, and bladder urothelial cancer. Later, these antibodies were also approved for renal cell carcinoma and squamous head and neck cancers. In addition, atezolizumab, which was initially approved for non-small cell lung cancer and bladder urothelial cancer, was also later approved for additional indications, namely small cell lung cancer, triple-negative breast cancer, and hepatocellular carcinoma. The treatment of other types of cancer by anti-PD-1/PD-L1 drugs alone, or in combination with immunotherapeutic and non-immunotherapeutic drugs, has also been approved more recently (4).

Due to the fact that immunotherapy treatment stimulates the natural immune defence against cancer cells, its adverse effects are related to immune responses of normal cells. Although anti-PD-1 drugs are overall less toxic than other oncologic treatments, such as standard chemotherapy, irAEs have been described in several clinical trials. For example, adverse effects related to organ-specific immune mechanisms have been described, including colitis, hepatitis, pneumonitis, and hypothyroidism, as well as general adverse events related to immune activation, including fatigue, diarrhoea, and dermatitis. Other less frequent adverse effects potentially attributable to immune mechanisms, such as musculoskeletal problems or neurologic alterations, have also been described in patients treated with immunotherapy. Although the real frequency of these rare adverse effects is not known, they may negatively impact a patient's quality of life, and so a better understanding of irAEs is necessary to determine the risk–benefit ratio for each patient when prescribing anti-PD-1/PD-L1 drugs (5).

Moreover, since treatment with anti-PD-1 or anti-PD-L1 therapies can require months or years to complete, it is also important to know the frequency of such adverse effects over the duration of treatment. Although these effects could appear at any time during treatment, it has been reported that those related to skin, gastrointestinal, and hepatic reactions tend to appear earlier than those related to the pulmonary, endocrine, and renal systems (6). Given that such information is scarce, physicians should be aware of how to manage patients who suffer from irAEs during treatment. Indeed, despite the relatively low rates of high-grade side effects with these treatments (usually ~10%), some can be life-threatening and require urgent and appropriate management (7). In addition, since immunotherapy treatment is being gradually expanded to patients with earlier-stage cancer and thus, longer life expectancies, the collection of such information becomes paramount (8).

Currently, the available information related to the management of immune toxicity is obtained from the meta-analysis of randomised clinical trials and from observational retrospective studies or case reports, but prospective information on the detection and management of the toxicity is lacking (4, 9, 10).

The aim of our study is therefore to characterise and describe the incidence of adverse reactions occurring in patients who began immunotherapy treatment in clinical practice at our institution, specifically focusing on those who underwent treatment with an anti-PD1/PDL1 monotherapy and focusing on the frequency of irADRs.

## Materials and methods

This prospective observational study evaluated cancer patients who were consecutively treated in real-world practice with monotherapy of PD-1/PD-L1 checkpoint inhibitors (standard schedules) from November 2017 to June 2019, wherein patient follow-up was continued until June 2021, regardless of the treatment line employed. The patients were followed until treatment interruption or until the end of the study. The study was conducted at the Vall d'Hebron University Hospital (Catalunya, Spain) and the study protocol was approved by the Ethical Committee of the same hospital (16/6/2017).

The inclusion criteria included patients ≥18 years of age who began treatment with nivolumab or pembrolizumab following a diagnosis of metastatic non-small cell lung cancer, advanced melanoma, or advanced renal cancer. Eight months after the study was commenced, an amendment was made to the protocol, and patients treated with atezolizumab were included. Other indications were also added to the protocol at this point, including squamous cell head and neck cancers, and urothelial carcinoma (advanced or metastatic). Patients treated with other immunotherapy drugs or with a combination of such drugs were excluded, as were those participating in clinical trials.

### Data sources

Patients were identified using the daily treatment list of the pharmacy service and were included after signing and documenting their informed consent. Each patient was contacted monthly by telephone and was interviewed in relation to the occurrence of adverse reactions. A structured questionnaire was employed for this purpose. The monthly telephone interview carried out to obtain information related to any adverse effects began with an open question, followed by some symptom-focused questions (e.g., related to organ-specific irADRs, including colitis, hepatitis, pneumonitis, and hypothyroidism, as well as more general adverse reactions related to immune activation, including fatigue, diarrhoea, and dermatitis). To obtain further information, the patient's electronic health record and laboratory test results (i.e., the results of blood tests, diagnostic imaging, or pathological anatomy assessments) were periodically reviewed. All patients were followed until 1 month after the end of treatment, irrespective of the reason for discontinuing treatment (e.g., disease progression, adverse effects, death, or other). However, some adverse effects were followed up until the end of the study to obtain further information regarding the treatment outcome. A total of 86 patients were included, thereby allowing us to estimate the ADR occurrence proportion with a precision of ±10%.

### Outcome measures

Information regarding the demographic and clinical variables was collected from the clinical medical records, as were variables related to the cancer (i.e., cancer type, date of diagnosis, stage of cancer upon commencing immunotherapy treatment, and number of previous treatment lines). Complete information related to the immunotherapy treatment employed and regarding other concomitant treatments was also gathered (i.e., type of drug, dosage, and the start/end treatment dates).

The primary outcome of our study was the characterisation of the ADRs experienced by cancer patients following the initiation of immune checkpoint therapy. The definition of ADRs used for the purpose of this study was as that stated in the European and Spanish regulations (11, 12). Literature data corresponding to immune-related adverse drug reactions (irADRs) were used to classify the ADRs as irADRs (8, 13, 14).

For each adverse effect, the onset date, severity, whether any additional treatment was required, and the outcome were registered. We used the Common Terminology of Clinical Adverse Events version 5.0 (CTCAE v5.0) of the Cancer National Institute categorisation to identify grades 3–5 as serious and grades 1–2 for all other reactions (15). In addition, the severity of each adverse effect was classified according to the European Union criteria (16). The MedDRA dictionary of medical terminology was used to classify the ADRs, while the drugs used for treatment were classified according to the Anatomical Chemical Classification (ATC) system (17, 18).

The imputability analysis of the drugs and the evaluation of any causal relationship between the drugs and the suspected adverse reactions were analysed using the methods and algorithm provided by the Spanish Pharmacovigilance System (SEFV) (19).

### Data analysis

The frequencies and incidences of all ADRs and irADRs were calculated during the study period. In addition, the ADR frequency was analysed by taking into account the following criteria: the affected organ/system, the reaction seriousness, whether the reaction was immune-related or late-onset immune-related, and the drug treatment employed.

The ADR outcomes were described along with the type of treatment and the reason for discontinuing treatment. For analysis of the ADR management protocol, four categories were considered: non-intervention or hygienic-dietetic measures, surgery, transfusion, and pharmacological measures.

The reaction frequencies and proportions were used for the descriptive analysis of the categorical variables, while the median, the Q1 and Q3 quartile values, and the minimum/maximum values were used for the continuous variables.

The ADR incidences were calculated by dividing the number of ADRs by the corresponding time in treatment and were expressed in cases per 100 patients per year (p-y) of exposure; the 95% confidence intervals were estimated from the Poisson distribution.

The analyses were performed using SAS^®^ 9.4 software (SAS Institute Inc., Cary, NC, United States).

## Results

### General patient characteristics

**Figure 1:**
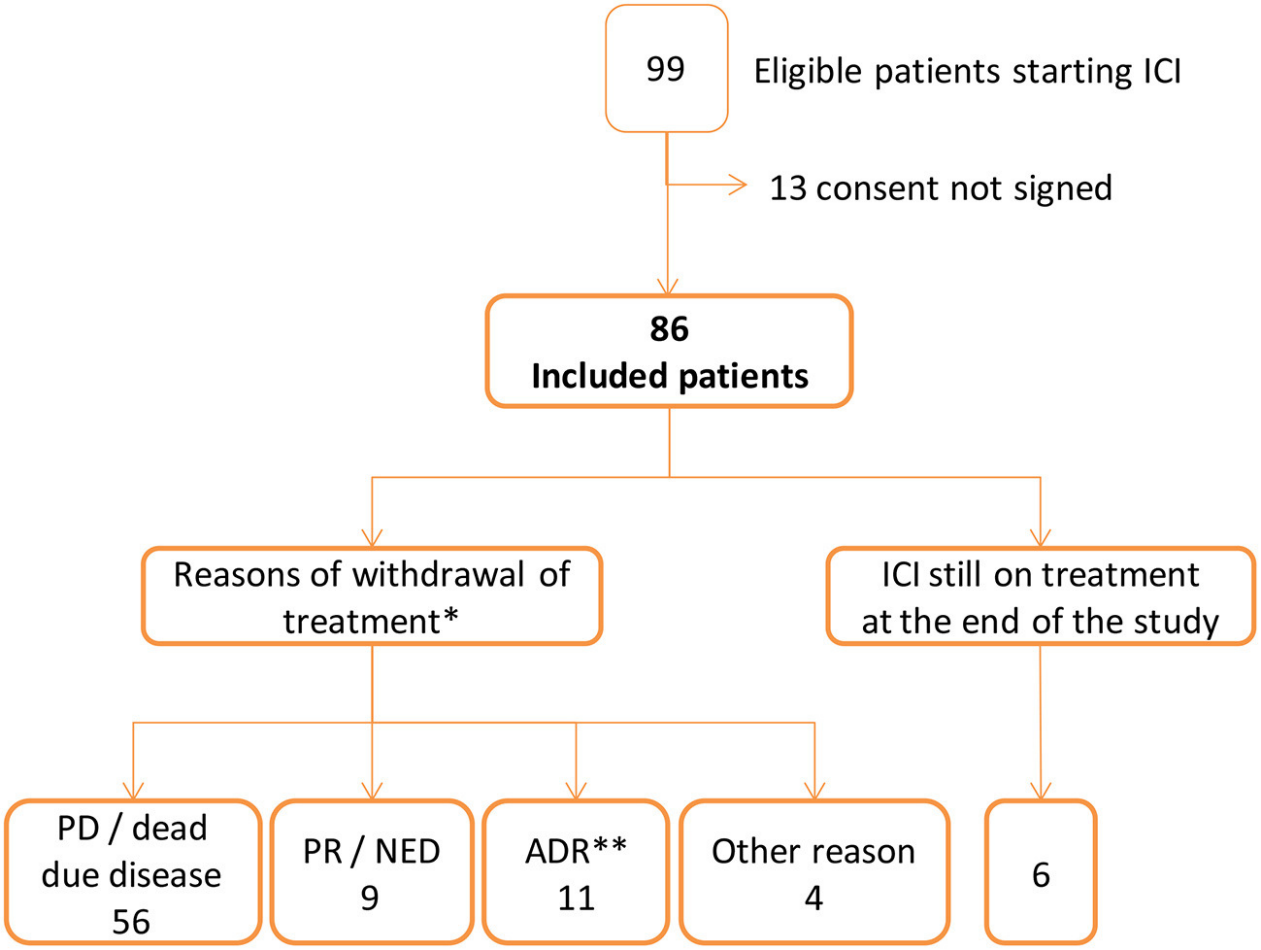
Flow chart of patients starting immune checkpoint inhibitors (ICI). *PD, progression disease; PR, partial response; NED, no-evidence disease; ADR, adverse drug reaction. **ADR was the only reason for ending treatment in 6 patients: adrenal insufficiency and cholestatic liver injury (1), acute renal insufficiency (1), interstitial pneumonitis (1), bipulmonary infiltrates (1), hypopituitarism (1), autoimmune colitis and cytomegalovirus gastrointestinal infection (1). In 5 patients there were additional reasons: pruritus and PD (1), adrenal insufficiency and PD (1), hyperamylasaemia and NED (1), dermatitis psoriasiform and rash and PR (1) and diarrhoea and PR (1). Other reasons of ending ICI treatment were appendicitis (1 patient), ictus (1), patient withdraw consent at the third visit (1), cognitive impairment identified at the second visit (1).

Out of the 99 patients identified, 86 met the inclusion criteria. The total cohort follow up was a median of 5.68 months (IQR: 2.8–20). The majority were male (67.4%), with a median age of 66 years (IQR: 59–76), and the median Charlson comorbidity index was 4 (IQR: 2–6). The most frequent cancer was non-small cellular lung cancer (46 cases, 53.5%), followed by melanoma (22 cases, 25.6%). Twenty-seven patients (31.4%) received a first line treatment (Figure 1, Table 1). Fourteen patients (16%) suffered from a locally advanced disease, and 72 (84%) exhibited metastasis.

**Table 1:** General characteristics of patients.

**Included patients, *n* (%)**	**NSCLC 46 (53.5)**	**Melanoma 22 (25.6)**	**Head and neck 9 (10.5)**	**Renovesical 9 (10.5)**	**Total 86 (100)**
Age, median (IQR) (min-max), years	66.5 (60–72) (41–87)	74 (63–80) (39–88)	57 (54–62) (50–65)	72 (58–78) (38–85)	66 (59–76) (38–88)
Gender, male/female, *n* (%)	30 (65.2)/16 (34.8)	12 (54.5)/10 (45.5)	8 (88.9)/1 (11.1)	8 (88.9)/1 (11.1)	58 (67.4)/28 (32.6)
Charlson CIS, median (IQR) (min-max)	5 (3–8) (2–14)	4 (2–6) (2–11)	3 (2–4) (2–5)	6 (3–8) (2–14)	4 (2–6) (2–14)
Treatment, 1^st^L / 2°*n*L or more, n (%)	7 (15.2)/39 (84.8)	17 (77.3)/5 (22.7)	3 (33.3)/6 (66.7)	0/9 (100)	27 (31.4)/59 (68.6)
Anti-PD-1	7 (15.2)/28 (60.9)	17 (77.3)/5 (22.7)	3 (33.3)/6 (66.7)	0/8 (88.9)	27 (31.4)/47 (54.7)
Anti-PD-L1	0/11(23.91)	0/0	0/0	0/1(11.11)	0/12 (14.0)
Duration of treatment, median (IQR) (min-max), months	2.7 (1.4–12.2) (0.0–33.4)	9.7 (3.5–11.5) (1.0–30.1)	15.2 (2.7–28.0) (0.5–39.3)	3.9 (2.8–24.2) (0.0–41.0)	4.9 (1.5–16.4) (0.0–41.0)
Anti-PD-1	2.4 (1.4–12.6) (0.0–33.4)	9.7 (3.5–11.5) (1.0–30.1)	15.2 (2.7–28.0) (0.5–39.3)	3.6 (2.6–22.1) (0.0–41.0)	4.9 (1.9–17.0) (0–41)
Anti-PD-L1	4.2 (1.0–8.2) (0.7–18.9)	-	-	31.3 (31.3–31.3) (31.3–31.3)	5.9 (1.2–12.3) (0.7–31.3)
Reasons to stop the treatment, *n* (%)^*^	45 (97.8)	21 (95.5)	7 (77.8)	7 (77.8)	80 (93.0)
PD/death	36 (80.0)	8 (38.1)	6 (85.7)	6 (85.7)	56 (70.0)
ADR	6 (13.3)	4 (19.1)	1 (14.3)	0	11 (13.8)
NED/PR	3 (6.7)	6 (28.6)	0	0	9 (11.3)
Other reasons^**^	0	3 (14.3)	0	1 (14.3)	4 (5.0)
Still on treatment at the end of the study, *n* (%)	1 (2.2)	1 (4.6)	2 (22.2)	2 (22.2)	6 (7.0)

Charlson Comorbidity Index Score (0–36).

* PD, Progression disease or death related to cancer; NED, no evidence of disease; PR, partial response; ADR, adverse drug reaction. ADR was the only cause of withdrawal in 6 patients.

** Other reasons: appendicitis (1), ictus (1), patient withdraw consent (1), cognitive impairment identified at the second visit (1).

NSCLC, Non-small cell lung cancer.

A total of 74 patients (86%) were treated with anti-PD-1 drugs and 12 (14%) were treated with anti-PD-L1 drugs. The median treatment durations were 4.9 (IQR: 1.9–17.0) and 5.9 months (IQR: 1.2–12.3), respectively (Table 1).

Treatment was stopped in 80 patients for the following reasons: i) 56 patients (70%), disease progression or death; ii) 11 patients (14%), the occurrence of an ADR; iii) 9 patients (11%), no evidence of disease (complete response) or a partial response; and iv) 4 patients (5%), other reasons. A total of 6 patients were still under treatment at the end of the study (median 31 months, IQR 27.9–33.4) (Figure 1, Table 1).

### Adverse drug reactions: Overall and immune-related reactions

During the follow-up, 75 patients (87.2%) were found to have reported a total of 353 ADRs, representing a global incidence of 215.5 ADRs/100 p-y following treatment (CI 95%: 194.2–239.2). Skin reactions (52 cases, 15%), general disorders (49 cases, 14%) (such as asthenia, fatigue andpyrexia), and gastrointestinal disorders (47 cases, 13%) were the most frequent (Table 2).

**Table 2:** Adverse drug reactions by system organ class disorders.

**System organ class**	**irADRs *n* (%)**	Patients^*^n (%)	**non irADRs *n* (%)**	Patients^*^n (%)	**All ADRs *n* (%)**	Patients^*^n (%)
**Skin and subcutaneous tissue**	50 (32.1)	34 (54.0)	2 (1.0)	2 (3.0)	52 (14.7)	36 (48.0)
Alopecia	1	1	0	0	1	1
Dermatitis psoriasiform	1	1	0	0	1	1
Dry skin	10	10	0	0	10	10
Eczema	1	1	0	0	1	1
Erythema	4	4	0	0	4	4
Exfoliative rash	1	1	0	0	1	1
Hyperhidrosis	0	0	1	1	1	1
Hyperkeratosis	1	1	0	0	1	1
Nail discolouration	1	1	0	0	1	1
Nail growth abnormal	1	1	0	0	1	1
Penile ulceration	1	1	0	0	1	1
Plantar erythema	1	1	0	0	1	1
Pruritus	16	16	0	0	16	16
Rash	5	5	0	0	5	5
Rash pruritic	2	2	0	0	2	2
Seborrhoeic dermatitis	1	1	0	1	1	2
Skin exfoliation	2	2	0	0	2	2
Vitiligo	1	1	0	0	1	1
**General and administration site cond**.	3 (1.9)	3 (4.8)	46 (23.4)	35 (53.0)	49 (13.9)	36 (48.0)
Asthenia	0	0	13	13	13	13
Fatigue	0	0	16	16	16	16
Feeling cold	0	0	3	3	3	3
Gait disturbance	0	0	1	1	1	1
Malaise	0	0	1	1	1	1
Mucosal dryness	3	3	0	0	3	3
Oedema peripheral	0	0	4	4	4	4
Pyrexia	0	0	7	7	7	7
Thirst	0	0	1	1	1	1
**Gastrointestinal**	29 (18.6)	21 (33.3)	18 (9.1)	15 (22.7)	47 (13.3)	33 (44.0)
Abdominal pain	0	0	2	2	2	2
Autoimmune colitis	1	1	0	0	1	1
Constipation	0	0	6	6	6	6
Dental dysaesthesia	0	0	1	1	1	1
Diarrhoea	13	12	0	0	13	12
Dry mouth	10	10	0	0	10	10
Lip oedema	0	0	2	1	2	1
Nausea	0	0	6	6	6	6
Stomatitis	5	5	0	0	5	5
Vomiting	0	0	1	1	1	1
**Infections and infestations**	1 (0.6)	1 (1.06)	39 (19.8)	25 (37.9)	40 (11.3)	25 (33.3)
Bronchitis	0	0	4	3	4	3
Campylobacter gastroenteritis	0	0	1	1	1	1
Conjunctivitis	0	0	2	2	2	2
Conjunctivitis viral	0	0	1	1	1	1
Cytomegalovirus gastrointestinal infection	0	0	1	1	1	1
Herpes zoster	0	0	1	1	1	1
Hordeolum	0	0	2	1	2	1
Influenza	0	0	1	1	1	1
Lower respiratory tract infection	0	0	1	1	1	1
Lower respiratory tract infection bacterial	0	0	1	1	1	1
Onychomycosis	0	0	1	1	1	1
Oral herpes	0	0	1	1	1	1
Oral infection	0	0	1	1	1	1
Other	0	0	1	1	1	1
Otitis externa	0	0	1	1	1	1
Peritonsillar abscess	0	0	1	1	1	1
Pneumonia	0	0	2	2	2	2
Respiratory tract infection	0	0	5	5	5	5
Rhinitis	1	1	0	0	1	1
Staphylococcal skin infection	0	0	1	1	1	1
Tooth abscess	0	0	1	1	1	1
Upper respiratory tract infection	0	0	4	3	4	3
Urinary tract infection	0	0	4	4	4	4
Urosepsis	0	0	1	1	1	1
**Metabolism and nutrition**	3 (1.9)	3 (4.8)	25 (12.7)	24 (36.4)	28 (7.9)	25 (33.3)
Abnormal loss of weight	0	0	2	2	2	2
Decreased appetite	0	0	14	14	14	14
Diabetic ketoacidosis	1	1	0	0	1	1
Hyperamylasaemia	2	2	0	0	2	2
Hypercholesterolaemia	0	0	3	3	3	3
Hyperkalaemia	0	0	2	2	2	2
Hypomagnesaemia	0	0	1	1	1	1
Hyponatraemia	0	0	1	1	1	1
Polydipsia	0	0	2	2	2	2
**Musculoskeletal and connective tissue**	17 (10.9)	16 (25.4)	5 (2.5)	5 (7.6)	22 (6.2)	18 (24.0)
Arthralgia	8	7	0	0	8	7
Bursitis	0	0	1	1	1	1
Muscle rigidity	0	0	1	1	1	1
Muscle spasms	0	0	2	2	2	2
Musculoskeletal pain	3	3	0	0	3	3
Myalgia	4	4	0	0	4	4
Osteonecrosis	0	0	1	1	1	1
Polyarthritis	1	1	0	0	1	1
Tendon pain	1	1	0	0	1	1
**Respiratory, thoracic and mediastinal**	10 (6.4)	8 (12.7)	12 (6.1)	11 (16.7)	22 (6.2)	17 (22.7)
Acute interstitial pneumonitis	1	1	0	0	1	1
Cough	0	0	5	5	5	5
Increased viscosity of upper respiratory secretion	0	0	1	1	1	1
Lung infiltration	1	1	0	0	1	1
Organising pneumonia	1	1	0	0	1	1
Pneumonitis	1	1	0	0	1	1
Productive cough	0	0	3	3	3	3
Pulmonary embolism	0	0	1	1	1	1
Respiratory failure	0	0	1	1	1	1
Rhinorrhoea	4	4	0	0	4	4
Suffocation feeling	0	0	1	1	1	1
Throat irritation	2	2	0	0	2	2
**Nervous system**	0	0	19 (9.6)	17 (25.8)	19 (5.4)	17 (22.7)
Balance disorder	0	0	1	1	1	1
Dizziness	0	0	1	1	1	1
Dysgeusia	0	0	3	3	3	3
Headache	0	0	3	3	3	3
Paraesthesia	0	0	9	9	9	9
Tonic clonic movements	0	0	1	1	1	1
Tremor	0	0	1	1	1	1
**Endocrine**	14 (9.0)	13 (20.6)	0	0	14 (4.0)	13 (17.3)
Adrenal insufficiency	6	6	0	0	6	6
Hypophysitis	1	1	0	0	1	1
Hypopituitarism	1	1	0	0	1	1
Hypothyroidism	6	6	0	0	6	6
**Eye**	12 (7.7)	8 (12.7)	2 (1.0)	2 (3.0)	14 (4.0)	10 (13.3)
Conjuctival hyperaemia	1	1	0	0	1	1
Corneal disorder	1	1	0	0	1	1
Corneal erosion	1	1	0	0	1	1
Dry eye	3	3	0	0	3	3
Eye pruritus	2	2	0	0	2	2
Eyelid cyst	1	1	0	0	1	1
Photophobia	1	1	0	0	1	1
Presbyopia	0	0	1	1	1	1
Vision blurred	2	2	0	0	2	2
Vitreous floaters	0	0	1	1	1	1
**Blood and lymphatic system**	3 (1.9)	3 (4.8)	11 (5.6)	7 (10.6)	14 (4.0)	10 (13.3)
Anaemia	0	0	6	6	6	6
Eosinophilia	2	2	0	0	2	2
Leukocytosis	0	0	1	1	1	1
Lymphopenia	0	0	2	2	2	2
Neutrophilia	0	0	1	1	1	1
Thrombocytopenia	1	1	0	0	1	1
Thrombocytosis	0	0	1	1	1	1
**Hepatobiliary**	12 (7.7)	10 (15.9)	0	0	12 (3.4)	10 (13.3)
Cholestasis	3	3	0	0	3	3
Cholestatic liver injury	6	6	0	0	6	6
Hepatocellular injury	3	2	0	0	3	2
**Renal and urinary**	2 (1.3)	2 (3.2)	6 (3.0)	6 (9.1)	8 (2.3)	8 (10.7)
Other	0	0	3	3	3	3
Renal failure	0	0	2	2	2	2
Renal impairment	1	1	0	0	1	1
Tubulointerstitial nephritis	1	1	0	0	1	1
Urinary incontinence	0	0	1	1	1	1
**Psychiatric**	0	0	6 (3.0)	6 (9.1)	6 (1.7)	6 (8.0)
Apathy	0	0	2	2	2	2
Depression	0	0	1	1	1	1
Depressive symptom	0	0	1	1	1	1
Other	0	0	1	1	1	1
Terminal insomnia	0	0	1	1	1	1
**Vascular**	0	0	5 (2.5)	5 (7.6)	5 (1.4)	5 (6.7)
Hypertension	0	0	3	3	3	3
Hypotension	0	0	1	1	1	1
Thrombophlebitis	0	0	1	1	1	1
**Neoplasms benign, malignant and NOS^a^**	0	0	1 (0.5)	1 (1.5)	1 (0.3)	1 (1.3)
Basal cell carcinoma	0	0	1	1	1	1
Total	156 (100)	63 (100)	197 (100)	66 (100)	353 (100)	75 (100)

* Patients may have more than one ADR.

a NOS, not otherwise specified; ADR, adverse drug reactions; irADR, immunorelated adverse drug reaction.

**Figure 2:**
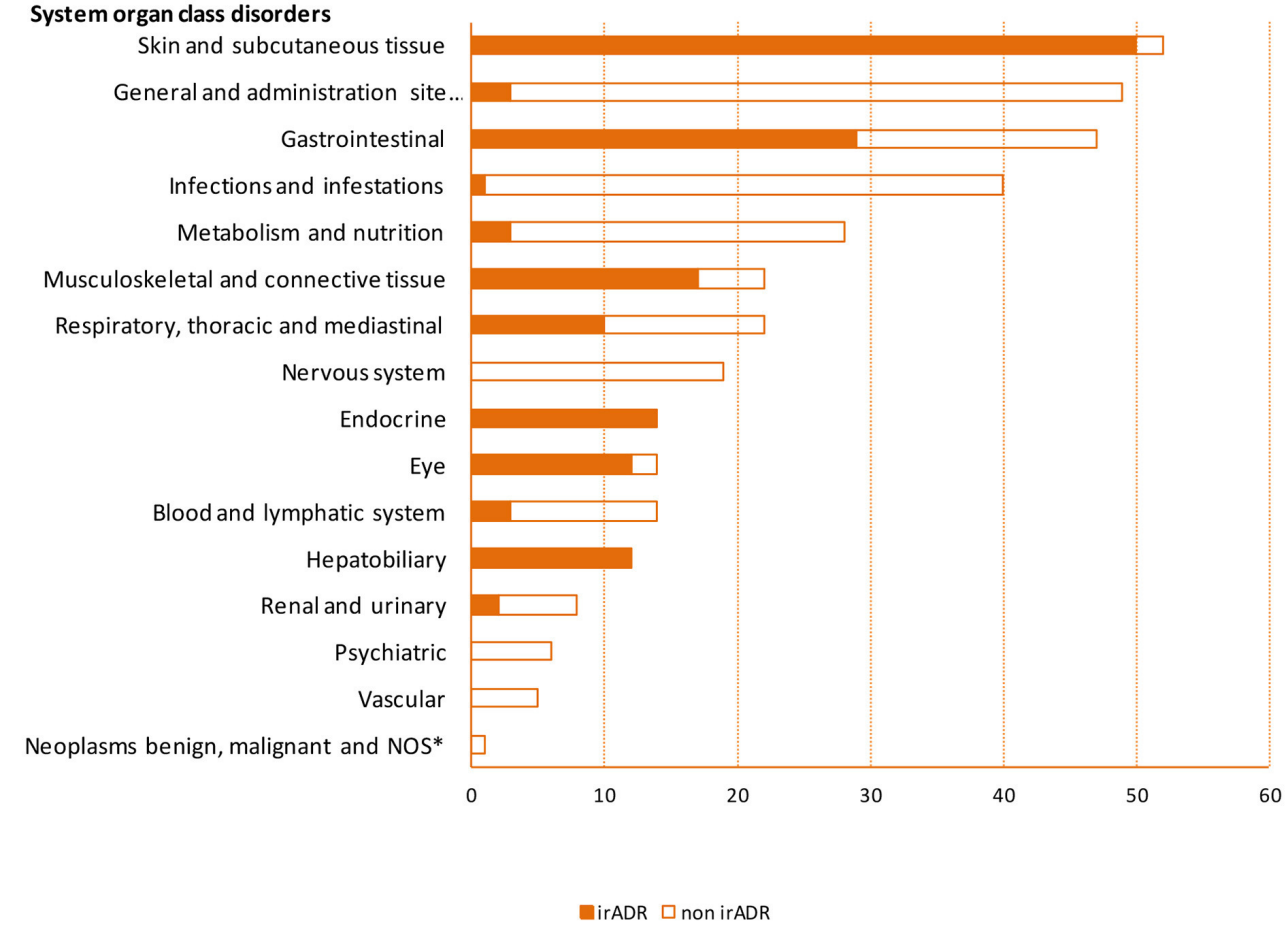
Frequency of adverse drug reactions by system organ class disorders. *NOS, not otherwise specified; irADR, immune-related adverse drug reaction.

In 63 patients (73%), a total of 156 (44% of the total number of ADR) irADRs were recorded, representing an incidence of 95.3 irADRs/100 p-y (CI 95%: 81.4–111.4). More specifically, skin disorders (50 cases, 32% of the 156 irADRs) with an incidence of 30.5 irADRs/100 p-y (23.1–40.3), gastrointestinal disorders (29 cases, 19%) with an incidence of 17.7 irADRs/100 p-y (12.3–25.5), musculoskeletal disorders (17 cases, 11%) with an incidence of 10.4 irADRs/100 p-y (6.5–16.70), and endocrine disorders (14 cases, 9%) with an incidence of 8.6 irADRs/100 p-y (5.1–14.4) were the most frequent (Figure 2, Supplementary Table S1).

**Figure 3:**
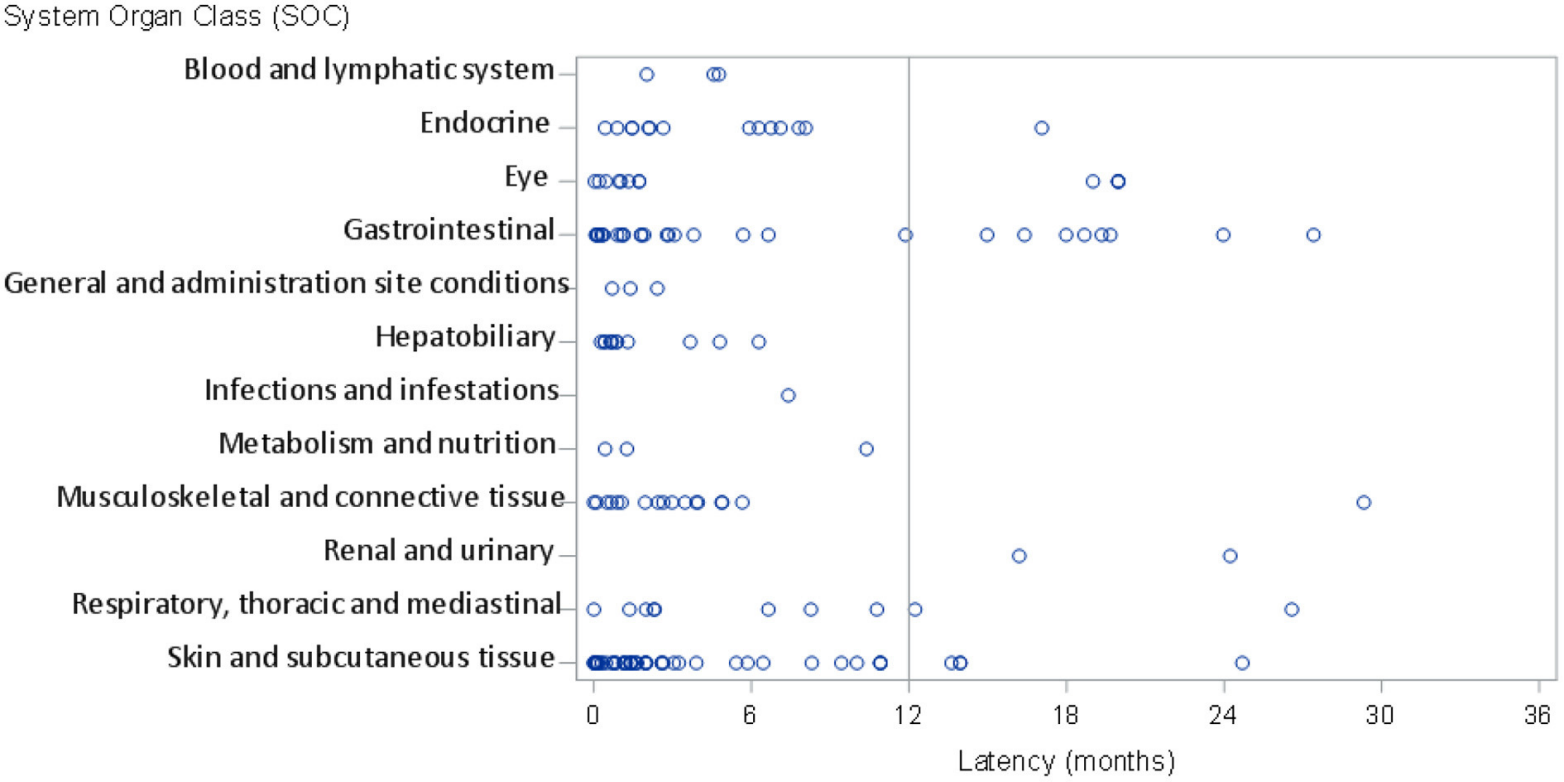
Latency period of immune-related adverse reactions.

Of the overall ADRs, in 45 cases (12% of the total number of ADRs) the latency period was ≥12 months, while a total of 22 irADRs (14% of the total number of irADRs) had a latency period of ≥12 months. Of these, 8 irADRs (27.6% of total gastrointestinal irADRs) affected the gastrointestinal system, 4 affected the eyes (33.3% of total eye irADRs), 4 affected skin and subcutaneous tissue (8% of total skin and subcutaneous irADRs) and 2 affected the renal and urinary system (100% of the renal and urinary system irADRs) (Figure 3, Supplementary Table S2). The detailed reactions, and all their characteristics are described in Table 3.

**Table 3:** Characteristics of late immune-related ADRs.

	**Severity**	**Management**
**Endocrine**		
Hypothyroidism	G1	Chronic treatment with Levothyroxine. Immunotherapy was continued.
**Eye**		
Conjunctival hyperaemia	G2	No treatment required. Immunotherapy withdrawn for another reason. Recovered
Corneal disorder	G1	Eye lubricating drops. Immunotherapy was continued. Recovered
Corneal erosion	G1	Ocular antibiotic treatment. Immunotherapy was continued. Recovered
Eyelid cyst	G2	Surgery. Immunotherapy was continued. Recovered
**Gastrointestinal**		
Autoimmune colitis	G5	Prednisone and infliximab treatment. Immunotherapy withdrawal. Death
Diarrhoea	G3	Serum therapy and antidiarrheal treatment. Immunotherapy was delayed for a week. Recovered
Diarrhoea	G3	Antidiarrheal treatment. Immunotherapy was continued. Recovered
Diarrhoea	G3	Serum and antidiarrheal treatment. Immunotherapy withdrawal for PD. Recovered
Diarrhoea	G2	Hygienic-dietetic measures. Immunotherapy was continued. Recovered
Diarrhoea	G2	Treatment with prednisone. Immunotherapy completed. Not recovered.
Diarrhoea	G1	Antidiarrheal treatment. Immunotherapy delayed for a week. Recovered
Stomatitis	G2	Nystatin treatment. Immunotherapy was continued. Recovered
**Musculoskeletal and connective tissue**		
Arthralgia	G1	No treatment required. Immunotherapy stopped for another reason. Recovered.
**Renal and urinary**		
Renal impairment	G1	Serum therapy. Immunotherapy continued. Recovered
Tubulointerstitial nephritis	G3	Prednisone treatment. Immunotherapy withdrawal for PD. Recovered
**Respiratory, thoracic and mediastinal**		
Lung infiltration	G2	No treatment required. Immunotherapy withdrawal. Recovered.
Pneumonitis	G1	No treatment required. Immunotherapy delayed for a week. Recovered.
**Skin and subcutaneous tissue**		
Dermatitis psoriasiform	G2	Prednisone and topical antiinfective treatment. Immunotherapy withdrawal for symptoms persistence. Recovered
Dry skin	G1	Hygienic-dietetic measures. Immunotherapy continued. Recovered
Pruritus	G1	Hygienic-dietetic measures. Immunotherapy continued. Recovered
Pruritus	G1	No treatment required. Immunotherapy withdrawal for another adverse drug reaction (worsening of renal impairment). Recovered

Regarding the seriousness of the reactions, 37 ADRs (10.5%) were categorized as grades 3–4, and 3 (1%) were categorized as grade 5 (death). Twelve of the irADRs (7.7%) were categorized as grades 3–4, and of these, 4 were diarrhoea, 3 were hepatocellular injuries, and the remainder consisted of one each of the following: diabetic ketoacidosis, hyperamylasaemia, polyarthritis, acute interstitial and tubulointerstitial nephritis, and pneumonitis. Only 2 irADRs (1.3%) were categorized as grade 5 (autoimmune colitis and hypopituitarism), the autoimmune colitis had a late presentation (>12 months) (Tables 3, 4, Supplementary Table S1).

**Table 4:** Adverse drug reactions, severity, and outcome.

**Adverse drug reactions n (%)**	**All ADRs 353 (100)**	**All irADRs 156 (44.47)**	**Early-irADRs 134 (100)**	**Late-irADRs 22 (100)**
ICI treatment				
Anti-PD-1	301 (85.3)	134 (85.9)	114 (85.1)	20 (90.9)
Anti-PD-L1	52 (14.7)	22 (14.1)	20 (14.9)	2 (9.1)
Severity of ADRs				
G1–G2	313 (88.7)	142 (91.0)	125 (93.3)	17 (77.3)
G3–G4	37 (10.5)	12 (7.7)	8 (5.9)	4 (18.2)
G5	3 (0.9)	2 (1.3)	1 (0.8)	1 (4.5)
Outcome				
Recovered	272 (77.1)	111 (71.2)	92 (82.9)	19 (17.1)
Recovering	27 (7.7)	19 (12.2)	17 (89.5)	2 (10.5)
Not recovered	46 (13.0)	23 (14.7)	23 (100)	0
Death	3 (0.9)	2 (1.3)	1 (50.0)	1 (50.0)
Unknown	5 (1.4)	1 (0.6)	1 (100)	0

ADRs, Adverse drug reactions; irADRs, immune-related ADRs; early-irADRs, immune-related ADRs with a latency period less than 12 months; late-irADRs, immune-related ADRs with a latency period equal or greater than 12 months.

In terms of ADR management, pharmacological treatment was required for 147 ADRs (41.7%) in 58 patients (77.3%), while 199 ADRs (56.5%) in 68 patients required no intervention or only hygienic-dietetic measures. In addition, 61 irADRs (39.1%) in 36 patients required pharmacological treatmen and, of these, 47 irADRs (30.1%) in 21 patients required treatment with corticosteroids, including oral and topical treatments. In only one case did the irADR require treatment with infliximab (Table 5, Supplementary Table S3). Additionally, in 13 patients treated with corticosteroids, it was necessary to interrupt the immunotherapy treatment; this interruption was permanent for 7 patients and temporary for the remainder.

**Table 5:** Management of adverse drug reactions.

***n* (%)**	All ADRs^*^353 (100)	**Patients 75 (100)**	**irADRs 156 (100)**	**Patients 63 (100)**
No intervention or hygienic-dietetic measures	199 (56.5)	68 (90.7)	94 (60.3)	50 (79.4)
Surgery treatment	3 (0.9)	3 (4.0)	1 (0.6)	1 (1.6)
Transfusion	3 (0.9)	3 (4.0)	0 (0)	0
Pharmacological measures^**^, *n* (%)	147 (41.7)	58 (77.3)	61 (39.1)	36 (57.1)
Analgesics	19	11	8	6
Antibacterials for systemic use	45	28	9	6
Antidiarrheals, intestinal antiinflammatory/antiinfective	15	10	15	10
Agents acting on the renin-angiotensin system	7	3	0	0
Antihistamines for systemic use	10	8	9	7
Antiinflammatory and antirheumatic products	7	5	5	3
Corticosteroids, dermatological preparations	11	7	10	6
Corticosteroids for systemic use	51	20	37	16
Diuretics	7	6	0	0
Drugs for obstructive airway diseases	6	3	0	0
Drugs for functional gastrointestinal disorders	9	8	1	1
Drugs for acid related disorders	5	4	0	0
Ophthalmologicals	12	5	3	2
Nasal preparations	5	4	3	2
Cough and cold preparations	5	5	0	0
Topical products for joint and muscular pain	5	3	4	3
Thyroid therapy	14	6	14	6
Others^***^	54	21	22	9

* Management was unknown in one ADR.

** Patients can be treated with one or more pharmacological measures; in 3 ADRs (1 patient) information on the specific drug was not available.

*** See details of other therapeutic groups with a frequency less than 5 on Supplementary Table S2.

In terms of the ADR outcome, in 272 of the total ADRs (77.1%), a “recovered” outcome was recorded, as was also the case for 111 (71.2%) of the irADRs. There were a total of 46 ADRs (13%) and 23 irADRs (14.7%) with a “non-recovered” outcome (Table 4).

## Discussion

In the present study we characterised the occurrence of ADRs, specifically irADRs, in cancer patients treated with immunotherapy in real-world clinical practice. The majority of patients experienced adverse reactions (87.2%), although most reactions were mild, with only 11.5% being categorized as grade 3 or above. A high percentage of the ADRs (44%) were immune-related, with skin disorders, gastrointestinal disorders, musculoskeletal disorders, and endocrine reactions being the most frequent. It is important to describe these results as they may have major implications for clinicians across multiple specialities who manage the rare, but clinically important, organ-specific irADRs.

In our study, the percentage of patients suffering from irADRs was found to be similar to that described by Nigro et al. in their retrospective study (76%), but higher than that reported by Majzoub et al. who quoted a figure of only 25% (9, 10). The criteria used for categorisation of the ADRs as immune-related based on the organ/system involved or through the literature identification, could explain these differences. In addition, intensive monitoring methods (monthly contact by telephone and structured interviews) were used in our study to identify patients suffering from irADRs, and such frequent contact could also account for the identification of greater numbers of affected patients. Regarding the severity of the irADRs, a similar percentage of irADRs was categorized as grade 3 or above in our study compared to that reported by Nigro et al. (i.e., 9.6%) (9). However, based on a meta-analysis involving 125 clinical trials, Y. Wang et al. reported that 14% of irADRs were grade 3 or above (20). These differences could be explained by considering the means by which the adverse reactions were selected, since in the above meta-analysis, all adverse events were gathered, whereas in our study, only the adverse drug reactions were evaluated.

Of the various irADRs described in the present study, the most frequent reactions were those affecting the skin, followed by general disorders, and those affecting the gastrointestinal system. These results are consistent with those described in the two retrospective studies and in both meta-analyses by Y. Wang et al. and P.F. Wang et al., wherein diarrhoea, colitis, and skin disorders were among the most frequently reported reactions (9, 10, 19, 21).

Importantly, it should be mentioned that although endocrinopathies associated with immunotherapy are not the most common irADRs reported in clinical trials, if they fail to be quickly and accurately recognised, they have the potential to become life-threatening. In this context, we note that a relatively high percentage and incidence of endocrine-related irADRs (i.e., 9%) were reported in our study, while in the meta-analysis by P.F. Wang et al., endocrine irADRs were reported for <2% of treated patients (20). Surprisingly, our data show that adrenal insufficiency and, hypothyroidism, were the most frequent endocrine-related irADR, with an incidence of 3.66 ADRs/100 p-y each. The occurrence of adrenal insufficiency was lower in the published meta-analysis by Y. Wang et al. (0.7%), and was not described in that published by Baxi et al. (4, 19). In our study, the information related to the diagnosis of adrenal insufficiency was collected from the medical records of patients; however, we cannot rule out the possibility that some of these cases were secondary to hypophysitis. Based on the above analyses, it is therefore apparent that intensive surveillance is necessary to diagnose these irADRs, and this is of particular importance since these cases may present with non-specific symptoms (8).

In terms of pneumonitis, we found a frequency of 3% when all presentations were included (i.e., interstitial pneumonitis, lung infiltration, and organised pneumonia). This proportion is similar to those reported in previous studies, such as in the meta-analysis by Y. Wang et al. (i.e., 2.8%) (19). However, we note that in a retrospective study by Majzoub et al., the percentage varied from 7.1% with nivolumab to 3.2% with ipilimumab (10). Despite its relatively low instance, pneumonitis is potentially life-threatening, and so surveillance is also necessary for this particular irADR. It should be noted here that for the purpose of our study, we did not include patients who had received combinations with ipilimumab.

Regarding the latency period, which is considered to be one of the areas of uncertainty, our data suggested that 14% of the irADRs appeared after 12 months from the start of treatment. However, Nigro et al. found that 30% of patients presented with a late-onset irADR. These differences may be due to the inclusion criteria employed in each study, since in the study by Nigro et al., only patients with a minimum treatment duration of 12 months were included. In contrast, in a high proportion of patients included in our study (65%), it was necessary to interrupt immunotherapy due to disease progression, thereby resulting in a shorter follow-up period for these patients. In both our study and in that by Nigro et al., it was found that a higher frequency of irADRs occurred in the early latency period rather than in the late one (9).

We also found that a high proportion of patients required some kind of pharmacological measure to treat their ADRs. In some cases, this was a permanent therapy replacement, as in the case of the endocrine irADRs. Such measures increase the complexity of patient management, in addition to resulting in a temporary or permanent interruption of their immunotherapy treatment. Furthermore, the need to administer corticosteroids in 21 (24%) patients and the necessity to interrupt treatment in 13 (15%) patients constitute lower numbers than those reported by Nigro et al., where 51 and 56% of patients suffering from early-onset irADRs and late-onset irADRs, respectively, required corticosteroid treatment, while 15.2 and 22% required their treatment to be interrupted (9). In our study, other pharmacological interventions for the treatment of diarrhoea, arthralgia, and other minor ADRs were also recorded, thereby resulting in higher percentages of patients receiving treatment.

The main strength of the present study is that it was specially designed to evaluate ADRs, and in particular, irADRs. The prospective nature of this study and the intensive monitoring of ADRs, along with the review of medical records and monthly phone calls to patients, allowed the comprehensive detection of ADRs. Moreover, the specific definition of an adverse reaction (11, 12) allowed us to rule out other concurrent events that were reported in previous studies. Furthermore, we systematically evaluated the causal relationships between the treatments employed and the suspected adverse reactions using the methods and algorithm provided by the Spanish Pharmacovigilance System (SEFV) (18). In addition, the long-term follow-up period and the specific attention paid to a variety of irADRs are expected to enhance our understanding of ADRs.

However, it should also be noted that some limitations can be found in our study. Firstly, it was a unicentric study, and the number of patients included was not sufficient to provide specific information related to each evaluated drug. However, a high percentage of the total cancer patients from throughout Catalunya attend our hospital. In addition, as the use of immunotherapy is increasing and the characteristics of treated patients may vary over time, the generalisability of our study could be affected. Furthermore, we did not evaluate combination therapies since the objective of this study was to monitor a unique active ingredient so as to avoid the contribution of other drugs when considering the attribution of causation. Finally, as mentioned above, we note that in a high proportion of patients, it was necessary to interrupt treatment due to disease progression, ultimately resulting in a short follow-up period for those patients.

## Conclusion

In our prospective observational study carried out at the Vall d'Hebron University Hospital (Catalunya, Spain), the majority of cancer patients treated with immunotherapy (i.e., monotherapy of PD1/PDL1 (programmed death-ligand 1) checkpoint inhibitors) experienced adverse drug reactions (ADRs). Although most reactions were mild, 11.5% were categorised as grade 3 or above. In addition, a high percentage of the ADRs were immune-related ADRs (irADRs) that occurred at any time during treatment, and therefore the early identification of such reactions through the close monitoring of patients is recommended. Indeed, the real-world data reported herein emphasise the requirement for the strict monitoring and multidisciplinary management of irADRs due to the fact that they often require pharmacological interventions, or could even be life-threatening. It is also possible that such irADRs could affect the continuation of immunotherapy treatment.

## Data availability statement

The raw data supporting the conclusions of this article will be made available by the authors, without undue reservation.

## Ethics statement

The studies involving human participants were reviewed and approved by Vall d'Hebron Hospital Ethical Committee. The patients/participants provided their written informed consent to participate in this study.

## Author contributions

MS, NG, M-JC, AF, EF, IB, JC, RM, and EM-C participated in the conception and design, interpretation of data, and writing the paper. EP participated in the conception and design, data analysis, interpretation of data, and writing the paper. XV participated in the design and data analysis. AA participated in the conception and design, interpretation of data, writing the paper, and coordinated the whole project. All authors contributed to the review and approved the final version of the manuscript.

## Conflict of interest

The authors declare that the research was conducted in the absence of any commercial or financial relationships that could be construed as a potential conflict of interest. The reviewer FMV declared a shared affiliation, with several of the authors MS, XV, and AA at the time of the review.

## Publisher's note

All claims expressed in this article are solely those of the authors and do not necessarily represent those of their affiliated organizations, or those of the publisher, the editors and the reviewers. Any product that may be evaluated in this article, or claim that may be made by its manufacturer, is not guaranteed or endorsed by the publisher.
