# Supplementary material for: Incidence and characteristics of adverse drug reactions in a cohort of patients treated with PD-1/PD-L1 inhibitors in real-world practice
Source: Front Med (Lausanne). 2022 Aug 22;9:891179. doi: 10.3389/fmed.2022.891179 (PMC9441693; doi:10.3389/fmed.2022.891179)
Supplement: Supplementary file 1 [file Data_Sheet_1.docx]

**Supplementary Table 1. Additional information of immune-related adverse drug reactions.**

|  | **Severity** | | | **irADR**  n (%) | **Incidence (95% CI)*** |
| --- | --- | --- | --- | --- | --- |
|  | G1-G2 | G3-G4 | G5 |  |  |
| **Blood and lymphatic system** | **3** | **0** | **0** | **3 (1.9)** | **1.83 (0.59-5.68)** |
| Eosinophilia | 2 | 0 | 0 | 2 | 1.22 (0.31-4.88) |
| Thrombocytopenia | 1 | 0 | 0 | 1 | 0.61 (0.09-4.33) |
| **Endocrine** | **13** | **0** | **1** | **14 (9.0)** | **8.55 (5.06-14.43)** |
| Adrenal insufficiency | 6 | 0 | 0 | 6 | 3.66 (1.65-8.15) |
| Hypophysitis | 1 | 0 | 0 | 1 | 0.61 (0.09-4.33) |
| Hypopituitarism | 0 | 0 | 1 | 1 | 0.61 (0.09-4.33) |
| Hypothyroidism | 6 | 0 | 0 | 6 | 3.66 (1.65-8.15) |
| **Eye** | **12** | **0** | **0** | **12 (7.7)** | **7.33 (4.16-12.90)** |
| Conjunctival hyperaemia | 1 | 0 | 0 | 1 | 0.61 (0.09-4.33) |
| Corneal disorder | 1 | 0 | 0 | 1 | 0.61 (0.09-4.33) |
| Corneal erosion | 1 | 0 | 0 | 1 | 0.61 (0.09-4.33) |
| Dry eye | 3 | 0 | 0 | 3 | 1.83 (0.09-4.33) |
| Eye pruritus | 2 | 0 | 0 | 2 | 1.22 (0.31-4.88) |
| Eyelid cyst | 1 | 0 | 0 | 1 | 0.61 (0.09-4.33) |
| Photophobia | 1 | 0 | 0 | 1 | 0.61 (0.09-4.33) |
| Vision blurred | 2 | 0 | 0 | 2 | 1.22 (0.31-4.88) |
| **Gastrointestinal** | **24** | **4** | **1** | **29 (18.6)** | **17.71 (12.31-25.48)** |
| Autoimmune colitis | 0 | 0 | 1 | 1 | 0.61 (0.09-4.33) |
| Diarrhoea | 9 | 4 | 0 | 13 | 7.94 (4.61-13.67) |
| Dry mouth | 10 | 0 | 0 | 10 | 6.11 (3.29-11.35) |
| Stomatitis | 5 | 0 | 0 | 5 | 3.05 (1.27-7.33) |
| **General and administration site conditions** | **3** | **0** | **0** | **3 (1.9)** | **1.83 (0.59-5.68)** |
| Mucosal dryness | 3 | 0 | 0 | 3 | 1.83 (0.59-5.68) |
| **Hepatobiliary** | **9** | **3** | **0** | **12 (7.7)** | **7.33 (4.16-12.90)** |
| Cholestasis | 3 | 0 | 0 | 3 | 1.83 (0.59-5.68) |
| Cholestatic liver injury | 6 | 0 | 0 | 6 | 3.66 (1.65-8.15) |
| Hepatocellular injury | 0 | 3 | 0 | 3 | 1.83 (0.59-5.68) |
| **Infections and infestations** | **1** | **0** | **0** | **1 (0.6)** | **0.61 (0.09-4.33)** |
| Rhinitis | 1 | 0 | 0 | 1 | 0.61 (0.09-4.33) |
| **Metabolism and nutrition** | **1** | **2** | **0** | **3 (1.9)** | **1.83 (0.59-5.68)** |
| Diabetic ketoacidosis | 0 | 1 | 0 | 1 | 0.61 (0.09-4.33) |
| Hyperamylasaemia | 1 | 1 | 0 | 2 | 1.22 (0.31-4.88) |
| **Musculoskeletal and connective tissue** | **16** | **1** | **0** | **17 (10.9)** | **10.38 (6.45-16.70)** |
| Arthralgia | 8 | 0 | 0 | 8 | 4.88 (2.44-9.77) |
| Musculoskeletal pain | 3 | 0 | 0 | 3 | 1.83 (0.59-5.68) |
| Myalgia | 4 | 0 | 0 | 4 | 2.44 (0.92-6.51) |
| Polyarthritis | 0 | 1 | 0 | 1 | 0.61 (0.09-4.33) |
| Tendon pain | 1 | 0 | 0 | 1 | 0.61 (0.09-4.33) |
| **Renal and urinary** | **1** | **1** | **0** | **2 (1.3)** | **1.22 (0.31-4.88)** |
| Renal impairment | 1 | 0 | 0 | 1 | 0.61 (0.09-4.33) |
| Tubulointerstitial nephritis | 0 | 1 | 0 | 1 | 0.61 (0.09-4.33) |
| **Respiratory, thoracic and mediastinal** | **9** | **1** | **0** | **10 (6.4)** | **6.11 (3.29-11.35)** |
| Acute interstitial pneumonitis | 0 | 1 | 0 | 1 | 0.61 (0.09-4.33) |
| Lung infiltration | 1 | 0 | 0 | 1 | 0.61 (0.09-4.33) |
| Organising pneumonia | 1 | 0 | 0 | 1 | 0.61 (0.09-4.33) |
| Pneumonitis | 1 | 0 | 0 | 1 | 0.61 (0.09-4.33) |
| Rhinorrhoea | 4 | 0 | 0 | 4 | 2.44 (0.92-6.51) |
| Throat irritation | 2 | 0 | 0 | 2 | 1.22 (0.31-4.88) |
| **Skin and subcutaneous tissue** | **50** | **0** | **0** | **50 (32.1)** | **30.53 (23.14-40.28)** |
| Alopecia | 1 | 0 | 0 | 1 | 0.61 (0.09-4.33) |
| Dermatitis psoriasiform | 1 | 0 | 0 | 1 | 0.61 (0.09-4.33) |
| Dry skin | 10 | 0 | 0 | 10 | 6.11 (3.29-11.35) |
| Eczema | 1 | 0 | 0 | 1 | 0.61 (0.09-4.33) |
| Erythema | 4 | 0 | 0 | 4 | 2.44 (0.92-6.51) |
| Exfoliative rash | 1 | 0 | 0 | 1 | 0.61 (0.09-4.33) |
| Hyperkeratosis | 1 | 0 | 0 | 1 | 0.61 (0.09-4.33) |
| Nail discolouration | 1 | 0 | 0 | 1 | 0.61 (0.09-4.33) |
| Nail growth abnormal | 1 | 0 | 0 | 1 | 0.61 (0.09-4.33) |
| Penile ulceration | 1 | 0 | 0 | 1 | 0.61 (0.09-4.33) |
| Plantar erythema | 1 | 0 | 0 | 1 | 0.61 (0.09-4.33) |
| Pruritus | 16 | 0 | 0 | 16 | 9.77 (5.99-15.95) |
| Rash | 5 | 0 | 0 | 5 | 3.05 (1.27-7.33) |
| Rash pruritic | 2 | 0 | 0 | 2 | 1.22 (0.31-4.88) |
| Seborrhoeic dermatitis | 1 | 0 | 0 | 1 | 0.61 (0.09-4.33) |
| Skin exfoliation | 2 | 0 | 0 | 2 | 1.22 (0.31-4.88) |
| Vitiligo | 1 | 0 | 0 | 1 | 0.61 (0.09-4.33) |

*incidence of irADRs: number of irADR per 100 patients-year of exposure of treatment and 95% of confidence interval.

**Supplementary Table 2. Details of latency period of immune-related adverse reactions.**

|  | **Latency (months)** | |
| --- | --- | --- |
| **System Organ Class (SOC)** | **Early-irADRs** | **Late-irADRs** |
| Blood and lymphatic system | 3 (100) | 0 |
| Endocrine | 13 (92.9) | 1 (7.1) |
| Eye | 8 (66.7) | 4 (33.3) |
| Gastrointestinal | 21 (72.4) | 8 (27.6) |
| General and administration site conditions | 3 (100) | 0 |
| Hepatobiliary | 12 (100) | 0 |
| Infections and infestations | 1 (100) | 0 |
| Metabolism and nutrition | 3 (100) | 0 |
| Musculoskeletal and connective tissue | 16 (94.1) | 1 (5.9) |
| Renal and urinary | 0 | 2 (100) |
| Respiratory, thoracic, and mediastinal | 8 (80.0) | 2 (20.0) |
| Skin and subcutaneous tissue | 46 (92.0) | 4 (8.0) |
| Total | 134 (85.9) | 22 (14.1) |

**Supplementary Table 3. Management of adverse drug reactions (additional information).**

|  | All ADRs  n=353* | irADRs  n=156 |
| --- | --- | --- |
| No intervention or hygienic-dietetic measures | 199 (56.5) | 94 (60.3) |
| Surgery treatment | 3 (0.9) | 1 (0.6) |
| Transfusion | 3 (0.9) | 0 (0) |
| Pharmacological measures**, n (%) | 147 (41.7) | 61 (39.1) |
| Agents acting on the renin-angiotensin system | 7 | 0 |
| All other therapeutic products | 2 | 0 |
| Analgesics | 19 | 8 |
| Anesthetics | 1 | 0 |
| Anti-acne preparations | 1 | 1 |
| Antianemics | 3 | 0 |
| Antibacterials for systemic use | 45 | 9 |
| Antibiotics and chemotherapeutics for dermatological use | 2 | 1 |
| Antidiarrheals, intestinal anti-inflammatory/anti-infective agents | 15 | 15 |
| Antiepileptics | 4 | 0 |
| Antifungals for systemic use | 2 | 1 |
| Antifungals for topical use | 3 | 2 |
| Antihistamines for systemic use | 10 | 9 |
| Anti-inflammatory and antirheumatic products | 7 | 5 |
| Antiprotozoals | 1 | 1 |
| Antithrombotic agents | 3 | 0 |
| Antivirals for systemic use | 3 | 1 |
| Calcium channel blockers | 1 | 0 |
| Cardiac therapy | 1 | 1 |
| Corticosteroids for systemic use | 51 | 37 |
| Corticosteroids, dermatological preparations | 11 | 10 |
| Cough and cold preparations | 5 | 0 |
| Diuretics | 7 | 0 |
| Drugs for acid related disorders | 5 | 0 |
| Drugs for constipation | 3 | 0 |
| Drugs for functional gastrointestinal disorders | 9 | 1 |
| Drugs for obstructive airway diseases | 6 | 0 |
| Drugs used in diabetes | 2 | 2 |
| Endocrine therapy | 1 | 1 |
| Immunosuppressants | 1 | 1 |
| Mineral supplements | 1 | 1 |
| Nasal preparations | 5 | 3 |
| Ophthalmologic | 12 | 3 |
| Other dermatological preparations | 1 | 1 |
| Other drugs for disorders of the musculoskeletal system | 3 | 2 |
| Otologists | 1 | 0 |
| Psychoanaleptics | 3 | 0 |
| Psycholeptics | 4 | 0 |
| Stomatological preparations | 3 | 2 |
| Thyroid therapy | 14 | 14 |
| Tonics | 1 | 1 |
| Topical products for joint and muscular pain | 5 | 4 |
| Vitamins | 3 | 3 |

*Management was unknown on one ADR.

**Patients can be treated with one or more pharmacological measures; on 3 ADRs (1 patient) the name of drug was unknown.
